# Linking neural mass signals and spike train statistics through point process and linear systems theory

**DOI:** 10.1186/1471-2202-14-S1-P330

**Published:** 2013-07-08

**Authors:** Moritz Deger, Arvind Kumar, Ad Aertsen, Stefan Rotter

**Affiliations:** 1 School of Life Sciences, Brain Mind Institute and School of Computer and Communication Sciences, École Polytechnique Fédérale de Lausanne, 1015 EPFL, Switzerland; 2 Bernstein Center Freiburg & Faculty of Biology, University of Freiburg, 79104 Freiburg, Germany

## 

The relation between neural mass signals, like local field potentials (LFP) or electro-encephalograms (EEG), and the spiking activity of neurons in a network is still poorly understood. Recently, linear temporal filters have been used to map multi-unit activity (MUA) to LFP signals recorded at the same electrode [1]. Similar kernels have been previously identified relating simulated network activity to the human EEG [2]. However, currently there are no theoretical/computational models to explain the form of these filters that map MUA to LFP or EEG.

Here we studied the relation between MUA and LFP in a minimal network model of the neocortex. Using simplified statistical models of neurons [3,4], the firing rate response of neuronal populations to time-dependent inputs can be characterized as that of a high pass filter. At the same time, the LFP recorded in the neocortex can be interpreted as a measure of the summated synaptic input to the population of nearby neurons [5], filtered by the neuronal membranes and the recurrent network [6]. Combining these various filter operations, we arrive at the forward model (LFP to MUA) of a band-pass filter, which can be inverted to predict the LFP from the MUA. Our results explain the form of the experimentally obtained kernels [1] and provide insight into the encoding of a stimulus by local neuronal populations. Furthermore, our theory explains characteristic properties of the neocortical LFP, solely based on effective neuronal refractoriness, membrane filtering and recurrent connectivity.
